# Supplementary material for: HIVprotI: an integrated web based platform for prediction and design of HIV proteins inhibitors
Source: J Cheminform. 2018 Mar 9;10:12. doi: 10.1186/s13321-018-0266-y (PMC5845081; doi:10.1186/s13321-018-0266-y)
Supplement: Supplementary file 2 — Additional file 2. Source code of HIVProtI web server. [file 13321_2018_266_MOESM2_ESM.zip › HIVprotI_Source-code/hivprotI/weka-3-6-6/doc/weka/core/neighboursearch/balltrees/BottomUpConstructor.html]

BottomUpConstructor


---


|  |  |  |
| --- | --- | --- |
| **Overview** \| **Package** \| **Class** \| **Tree** \| **Deprecated** \| **Index** \| **Help** \| **Weka's home** | |  |
| **PREV CLASS**   **NEXT CLASS** | **FRAMES**    **NO FRAMES**     **All Classes** |  |
| SUMMARY: NESTED \| FIELD \| CONSTR \| METHOD | DETAIL: FIELD \| CONSTR \| METHOD |  |


---


## weka.core.neighboursearch.balltrees Class BottomUpConstructor

```
java.lang.Object
  weka.core.neighboursearch.balltrees.BallTreeConstructor
      weka.core.neighboursearch.balltrees.BottomUpConstructor
```

**All Implemented Interfaces:**: java.io.Serializable, OptionHandler, RevisionHandler, TechnicalInformationHandler

---

``` public class BottomUpConstructor extends BallTreeConstructor implements TechnicalInformationHandler ```

The class that constructs a ball tree bottom up.


BibTeX:

```
 @techreport{Omohundro1989,
    author = {Stephen M. Omohundro},
    institution = {International Computer Science Institute},
    month = {December},
    number = {TR-89-063},
    title = {Five Balltree Construction Algorithms},
    year = {1989}
 }
```


Valid options are:

```
 -N <value>
  Set maximum number of instances in a leaf node
  (default: 40)
```

```
 -R
  Set internal nodes' radius to the sum 
  of the child balls radii. So that it 
 contains the child balls.
```

**Version:**
:   $Revision: 1.3 $

**Author:**
:   Ashraf M. Kibriya (amk14[at-the-rate]cs[dot]waikato[dot]ac[dot]nz)

**See Also:**: Serialized Form

---

**Constructor Summary**

`BottomUpConstructor()`
  
          Creates a new instance of BottomUpConstructor.


| **Method Summary** | |
| --- | --- |
| `int[]` | `addInstance(BallNode node, Instance inst)`             Adds an instance to the ball tree. |
| `BallNode` | `buildTree()`             Builds the ball tree bottom up. |
| `Instance` | `calcPivot(weka.core.neighboursearch.balltrees.BottomUpConstructor.TempNode node1, weka.core.neighboursearch.balltrees.BottomUpConstructor.TempNode node2, Instances insts)`             Calculates the centroid pivot of a node based on its two child nodes. |
| `double` | `calcRadius(weka.core.neighboursearch.balltrees.BottomUpConstructor.TempNode n1, weka.core.neighboursearch.balltrees.BottomUpConstructor.TempNode n2)`             Calculates the radius of a node based on its two child nodes. |
| `java.lang.String` | `getRevision()`             Returns the revision string. |
| `TechnicalInformation` | `getTechnicalInformation()`             Returns an instance of a TechnicalInformation object, containing detailed information about the technical background of this class, e.g., paper reference or book this class is based on. |
| `java.lang.String` | `globalInfo()`             Returns a string describing this nearest neighbour search algorithm. |

**Methods inherited from class weka.core.neighboursearch.balltrees.BallTreeConstructor**

`containChildBallsTipText, getContainChildBalls, getMaxDepth, getMaxInstancesInLeaf, getMaxRelativeLeafRadius, getNumLeaves, getNumNodes, getOptions, listOptions, maxInstancesInLeafTipText, maxRelativeLeafRadiusTipText, setContainChildBalls, setEuclideanDistanceFunction, setInstanceList, setInstances, setMaxInstancesInLeaf, setMaxRelativeLeafRadius, setOptions`

**Methods inherited from class java.lang.Object**

`equals, getClass, hashCode, notify, notifyAll, toString, wait, wait, wait`

**Constructor Detail**

### BottomUpConstructor

```
public BottomUpConstructor()
```

:   Creates a new instance of BottomUpConstructor.


**Method Detail**

### globalInfo

```
public java.lang.String globalInfo()
```

:   Returns a string describing this nearest neighbour search algorithm.

    :   **Returns:**: a description of the algorithm for displaying in the explorer/experimenter gui

---


### getTechnicalInformation

```
public TechnicalInformation getTechnicalInformation()
```

:   Returns an instance of a TechnicalInformation object, containing detailed
    information about the technical background of this class, e.g., paper
    reference or book this class is based on.

    :   **Specified by:**: `getTechnicalInformation` in interface `TechnicalInformationHandler`
    :   **Returns:**: the technical information about this class

---


### buildTree

```
public BallNode buildTree()
                   throws java.lang.Exception
```

:   Builds the ball tree bottom up.

    :   **Specified by:**: `buildTree` in class `BallTreeConstructor`
    :   **Returns:**: The root node of the tree. **Throws:**: `java.lang.Exception` - If there is problem building the tree.

---


### addInstance

```
public int[] addInstance(BallNode node,
                         Instance inst)
                  throws java.lang.Exception
```

:   Adds an instance to the ball tree.

    :   **Specified by:**: `addInstance` in class `BallTreeConstructor`
    :   **Parameters:**: `node` - The root node of the tree.: `inst` - The instance to add to the tree. **Returns:**: The new master index array after adding the instance. **Throws:**: `java.lang.Exception` - Always as BottomUpConstructor does not allow addition of instances after batch construction.

---


### calcPivot

```
public Instance calcPivot(weka.core.neighboursearch.balltrees.BottomUpConstructor.TempNode node1,
                          weka.core.neighboursearch.balltrees.BottomUpConstructor.TempNode node2,
                          Instances insts)
                   throws java.lang.Exception
```

:   Calculates the centroid pivot of a node based on its
    two child nodes.

    :   **Parameters:**: `node1` - The first child node.: `node2` - The second child node.: `insts` - The instance on which the tree is to be built. **Returns:**: The centre/pivot of the node. **Throws:**: `java.lang.Exception` - If there is some problem calculating the centre/pivot of the node.

---


### calcRadius

```
public double calcRadius(weka.core.neighboursearch.balltrees.BottomUpConstructor.TempNode n1,
                         weka.core.neighboursearch.balltrees.BottomUpConstructor.TempNode n2)
                  throws java.lang.Exception
```

:   Calculates the radius of a node based on its two
    child nodes.

    :   **Parameters:**: `n1` - The first child node.: `n2` - The second child node. **Returns:**: The calculated radius of the the node. **Throws:**: `java.lang.Exception` - If there is some problem in calculating the radius.

---


### getRevision

```
public java.lang.String getRevision()
```

:   Returns the revision string.

    :   **Specified by:**: `getRevision` in interface `RevisionHandler` **Overrides:**: `getRevision` in class `BallTreeConstructor`
    :   **Returns:**: the revision


---


|  |  |  |
| --- | --- | --- |
| **Overview** \| **Package** \| **Class** \| **Tree** \| **Deprecated** \| **Index** \| **Help** \| **Weka's home** | |  |
| **PREV CLASS**   **NEXT CLASS** | **FRAMES**    **NO FRAMES**     **All Classes** |  |
| SUMMARY: NESTED \| FIELD \| CONSTR \| METHOD | DETAIL: FIELD \| CONSTR \| METHOD |  |


---
